# Effect of Prior Symptomatic Dengue Infection on Dengue Haemorrhagic Fever (DHF) in Children

**DOI:** 10.1155/2021/8842799

**Published:** 2021-06-02

**Authors:** Randula Ranawaka, Chamara Jayamanne, Kavinda Dayasiri, Dinuka Samaranayake, Udara Sandakelum, Wathsala Hathagoda, Ruwangi Dissanayake, Pujitha Wickramasinghe

**Affiliations:** ^1^Department of Paediatrics, Faculty of Medicine, University of Colombo, Colombo, Sri Lanka; ^2^University Paediatric Unit, Lady Ridgeway Hospital for Children, Colombo, Sri Lanka

## Abstract

Pathogenesis of dengue haemorrhagic fever is not fully understood, but it is thought that there is antibody enhancement during the secondary infection, which causes severe dengue haemorrhagic fever (DHF). Therefore, patients who have DHF should have a documented history of symptomatic dengue infection in the past. A retrospective descriptive-analytical study was conducted at the University Paediatric Unit at Lady Ridgeway Hospital for Children, Colombo, Sri Lanka. All children who had fulfilled the criteria for DHF admitted to the unit from April 2018 to September 2018 were recruited into the study. Relevant data were collected from bed head tickets. One hundred and eighty-four children were included in the final analysis. Thirty-three (17.9%) had a past history of documented symptomatic dengue infection, while 82.1% did not have a documented dengue infection. Twelve patients had dengue shock syndrome, and none of them had previously documented symptomatic dengue fever. Dextran was used in 96 patients in the critical phase. Twelve (42%) patients with past documented symptomatic dengue fever needed dextran while 84 (54.9%) patients without a documented past history of dengue fever needed dextran. In our clinical observation, we noticed that children with DHF mostly did not have a documented symptomatic prior dengue infection, while those with a documented symptomatic prior infection had a milder subsequent illness. In fact, the majority (82.1%) of patients with DHF did not have documented previous symptomatic dengue infection. It was also observed that the clinical course of subsequent dengue infection was less severe in patients with previously documented symptomatic dengue fever. This finding should be further evaluated in a larger scale study minimizing the all-confounding factors. This fact is more important in selecting recipients for vaccines against the dengue virus, which are supposed to produce immunity against the virus without causing the severe disease.

## 1. Introduction

Dengue is one of the fastest spreading mosquito-borne viral infections [1] and an important but neglected tropical disease [2]. Approximately, 50 million dengue infections occur annually in dengue-endemic countries [3]. Over 70% of total dengue infections are reported from Asia [4] whilst Western Pacific region and several countries in Latin America are other mainly affected geographic regions. Worldwide, the case fatality rate of dengue is approximately 1% [5]. However, left untreated mortality can be as high as 20% [6]. Sri Lanka experiences large outbreaks of dengue infection annually [7].

Presentation of dengue infection may be a spectrum ranging from asymptomatic to dengue fever (DF), dengue haemorrhagic fever (DHF) and to dengue shock syndrome [8]. Pathogenesis of dengue haemorrhagic fever is not fully understood, but it is widely accepted that there is an antibody-dependent enhancement (ADE) during the secondary infection which causes DHF [9]. ADE promotes immune reaction during the second encounter where the virus is present to target macrophages and monocytes more effectively triggering off severe infection and leading to develop DHF [10, 11].

Most of the primary dengue infections are asymptomatic [12–16]. According to the theory of antibody enhancement, patients will be more symptomatic during a subsequent attack of dengue infection irrespective of the symptoms and signs of the primary infection of dengue [17–19]. In our clinical observation, we noticed that majority of patients who had DHF did not have any documented symptomatic prior dengue infection and patients with prior symptomatic dengue infection had less severe disease. Thus, we hypothesized that prior symptomatic dengue infection will have a protective antibody formation that will in turn have significant protection over all serotypes of dengue in subsequent infection and could prevent the development of complications.

Moreover, according to the same hypothesis, a proposed live attenuated dengue vaccine could act as an asymptomatic infection in unexposed individuals, which could lead to the development of severe infection following the first wild-type infection according to our hypothesis. This safety issue was observed in the field testing of the recombinant live attenuated tetravalent CYD-TDV vaccine (Dengvaxia®, Sanofi Pasteur, France), where in 2- to 5-year-old Asian children, the vaccine group developed dengue infection requiring hospitalization than their control counterparts. This made the manufacturers recommend the vaccine for children above 9 years [20]. Thus, the results of this study may be of clinical value in deciding criteria for the selection of recipients to receive the vaccination to prevent dengue infection.

Therefore, this retrospective study was designed to assess the effect of past symptomatic dengue infection on the severity of DHF.

## 2. Methodology

A retrospective descriptive study was designed to assess these objectives at the Professorial Paediatric Unit, University of Colombo at Lady Ridgeway Hospital, Sri Lanka. All patients admitted to the unit from April 2018 till September 2018 with objectively proven DHF were included in the study.

Medical records of patients with DHF were retrospectively analyzed to find out whether they had any previous episodes of documented symptomatic dengue infection. Records without clear documentation regarding the presence or absence of previous dengue infection were not considered for the analysis. The case definition of DHF was based on the presence of high fever; haemorrhagic manifestations defined by a positive tourniquet test, oral or evidence of gastrointestinal bleed; thrombocytopenia of ≤100 × 10^9^/L; and objective evidence of capillary leak detected by ultrasound scan or clinical evidence of pleural effusion or peritoneal leak.

Data that were collected from the medical records were the age, sex, clinical symptoms of current illness, evidence of past infection of symptomatic dengue infection, complications that were developed during hospital stay, and requirement of fluids.

Information was verified with parents where information regarding previous infections was not documented clearly in records by contacting them over the phone. If they carried a diagnosis made by a medical person, clinically or serologically, was taken as evidence of past symptomatic dengue infection.

The severity of DHF was evaluated in relation to the presence of clinical shock and the need for Dextran boluses. The ward has a standard management protocol developed based on the national DF/DHF management guidelines for children and adolescents [21].

Data were analyzed using SPSS v20 (SPSS Inc., Chicago, IL, USA) statistical software package. Ethical approval was taken (date: 12/01/2018; Ref no. LRH/ERC/07/18) from the Ethics Review Committee of Lady Ridgeway Hospital, Colombo. Informed written consent was obtained from parents of participating children prior to their recruitment to the study.

## 3. Results

Data of 200 children who were diagnosed to have DHF during the study period were reviewed. 16 children were excluded due to inadequate documentation in medical records and data of 184 children were available for analysis. 94 (51.1%) were male children and there was no gender predisposition (*p* < 0.05).  Table 1 demonstrates the age and sex distribution of the study sample.

All children who were included had their leaking phase in DHF confirmed by a point-of-care inward ultrasound scan performed by a trained medical officer. Most children (167, 90.7%) had a platelet count of less than 100 × 10^9^/L at the onset of the leaking phase. The most common symptoms in the prodromal phase were vomiting (125, 67.9%), abdominal pain (121, 65.7%), and headache (112, 60.9%). The majority of children (136, 73.9%) had onset of critical phase while being managed at hospital. However, some children (48, 26.1%) presented to the hospital with evidence of leakage at different times of the critical phase varying from 06 hours to 36 hours from the time of onset.

**Figure 1:**
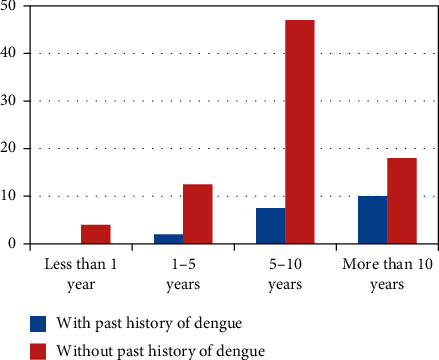
Distribution of symptomatic (with past history) and asymptomatic (without past history) dengue infections in children with DHF, according to age.

Thirty-three (17.9%) patients had a past history of documented symptomatic dengue while 151 children (82.1%) did not have documented dengue.  Figure 1 shows the age-wise distribution of previous symptomatic and asymptomatic dengue infections among children who had DHF.

Twelve patients presented with dengue shock syndrome and none of them had previously documented dengue infection (*p*=0.002). Dextran was used in 96 patients during the critical phase. Twelve (36.4%) patients with previously documented symptomatic dengue needed dextran while 84 (55.6%) patients without a past history of documented symptomatic dengue needed dextran in the management (*p* > 0.05).

## 4. Discussion

The pathophysiology of dengue haemorrhagic fever remains obscure, but it is thought to be mediated by antibody-dependent enhancement during the second infection from a different subtype of dengue virus [2]. It can be theoretically postulated that the subsequent infection from the dengue virus would be more severe than the first episode. Observations in the current study revealed that the prior asymptomatic dengue infection caused much severe disease than prior symptomatic infection, thus suggesting a potential immunity-related advantage in those with prior symptomatic infection over those who were asymptomatic in their previous infection. Currently, only a little evidence exists on this observation and the outcome of secondary infection likely depends on the nature of the immune response developed during primary dengue infection [22].

During the study, we found that all the patients who were admitted to Dengue High Dependency Unit with features of shock did not have previous symptomatic dengue infection. And also it clearly showed that the number of children who required dextran for circulatory support was higher in patients with no history of prior symptomatic infection although it did not show a statistical significance. One reason why a significant association was not seen could be due to the reduced sample size in the current study.

Previous epidemiological studies from South-East Asia have reported the burden of asymptomatic and in-apparent dengue infection and the impact of these infections in the causation of severe dengue during a second encounter [23]. The reverse has also been observed where the presence of more severe infections at one time can lead to only milder infections in a subsequent dengue encounter in the same person [24]. The epidemiological patterns and postulated pathophysiological mechanisms support potential immunity-related beneficial effects of symptomatic dengue infections over in-apparent or asymptomatic infections.

The findings of the current study are limited by the confounding effects of host (genetic factors, preexisting immunity for dengue) and viral virulence-related factors (serotype, genetic factors) and the nature of dengue transmission in the studied community. Even though our study population is small, it clearly showed the difference between the two groups supporting the theory of protective immunity from symptomatic infections over asymptomatic infections. This is an important observation regarding the severity of DHF in which the pathophysiology could be determined based on the nature of the immune response a person would develop following an encounter with dengue for the first time. Further studies are needed to accurately understand the nature of this immunopathophysiology.

The study also provides insights into the process of introducing an efficacious dengue vaccine. Despite dengue being a significant health problem in most parts of the world, there is no effective and safe vaccine which can provide long-term protection to date [21]. One of the main obstacles in developing an efficacious vaccine against dengue is the potential risk of antibody enhancement and occurrence of clinically significant disease with the subsequent infection, even it being the first infection [25–27]. In this background, it is important that an effective vaccine should trigger the production of cross-protective neutralizing, heterotypic antibodies rather than triggering off antibody-mediated enhancement or cell-mediated immunity.

Currently, the only FDA (Food and Drug Administration, USA) approved dengue vaccine is Dengvaxia (live attenuated tetravalent vaccine) and it is registered in twenty countries where dengue is endemic [28]. Although clinical trials on safety and immunogenicity of dengue vaccine [25] have shown type-specific neutralizing antibody responses which are superior to the controls, vaccine efficacy against any dengue serotype was low to moderate, suggesting lack of cross-protection and risk for DHF. Furthermore, its susceptibility to cause more hospitalized dengue infection in younger children supports our hypothesis [19]. It can be assumed that vaccine-induced immune response may have a similar effect compared to an asymptomatic dengue infection giving rise to the risk of DHF in subsequent dengue infection. For this reason, the dengue vaccine is not approved by FDA for those not previously infected by any dengue serotype or in whom serological status is unknown [29]. More robust studies are needed to understand the kinetics of and immune responses to past dengue infection and vaccines to ensure long-term heterotypic immune protection.

## 5. Conclusion

The majority of patients (82.1%) with DHF in the current study had no previously documented symptomatic episode of dengue infection. It seems that the course of illness is less severe in patients with previously documented symptomatic dengue fever. This finding should be further evaluated in a larger scale study minimizing the all-confounding factors. This fact is more important in selecting recipients for vaccines against the dengue virus, which are supposed to produce immunity against the virus without causing the severe disease.

## Figures and Tables

**Table 1:** Age and sex distribution of the study sample.

	Frequency	Percentage
Sex		
Male	94	51.1
Female	90	48.9

Age categories		
<1 year	7	3.8
1–5 years	26	14.1
>5–10 years	100	54.3
>10–14 years	51	27.7

## Data Availability

The data that support the findings of this study are available from Medical Records Department, Lady Ridgeway Children's Hospital, Colombo, but restrictions apply to the availability of these data, which were used under license for the current report and so are not publicly available. Data are, however, available from the authors upon reasonable request and with permission of the Medical Records Department, Lady Ridgeway Children's Hospital, Colombo, Sri Lanka.
